# Clinical outcome of short daily hemodialysis in the elderly

**DOI:** 10.1007/s40620-021-01148-1

**Published:** 2021-08-28

**Authors:** Patricia Villié, Maxime Dauvergne, Catherine Maheas, Florence Vendé, Pablo Urena, Christophe Ridel, Maxime Touzot

**Affiliations:** 1 Service de Dialyse et Aphérèse Thérapeutique, AURA, 75014 Paris, France; 2 Centre de Dialyse AURA Saint-Ouen, Saint-Ouen, France

**Keywords:** Daily dialysis, Elderly, Survival

Recent results from the frequent hemodialysis network (FHN) daily trial confirmed the clinical and biological benefits of in-center short daily hemodialysis (SDHD) compared to conventional thrice-weekly hemodialysis (HD) [1]. SDHD improves cardiovascular outcome, with better fluid volume balance as well as blood pressure and phosphate control, and moreover, it results in significantly longer survival after 2 years of treatment. However, this trial focused on fairly young patients, mainly aged under 50. Clinicians may consider it useful to treat elderly patients with a softer technique such as SDHD because of their numerous comorbidities.

In the present study, we report a retrospective assessment of 37 HD patients who were switched from conventional thrice-weekly HD to in-center SDHD. They were divided into two groups according to the median age of 65 years: SDHD < 65 (N = 19) and SDHD ≥ 65 (N = 18). SDHD was initiated at the patient’s request in 58% of the SDHD < 65 cohort, and by medical decision in 78% of the SDHD ≥ 65 group. Demographic characteristics at baseline are shown in Table S1. SDHD ≥ 65 had more cardiovascular diseases.

Interdialytic weight gain (IDWG) was significantly reduced by SDHD in all patients (Fig. S1), but to a greater extent in the SDHD ≥ 65 group (0.9 ± 0.6 vs 1.9 ± 0.9 kg, p < 0.01). After 12 months of follow-up, total weekly IDWG decreased slightly in SDHD ≥ 65 to 5.1 ± 3.6 vs 7.2 ± 5.5 kg but remained stable in SDHD < 65 (8 ± 5.1 vs 7.6 ± 4.9 kg), without statistical significance. Data regarding urine volume were not available. The dry weight remained stable in both groups during the follow-up.

Pre-dialysis systolic blood pressure (pre-HD sBP) was higher for SDHD < 65 compared to SDHD ≥ 65 during the follow-up: 141 ± 20 vs 127 ± 23 mmHg (*p* = 0.005), and 136 ± 21 vs 121 ± 28 mmHg (*p* = 0.015) (Fig. S2A). A similar trend was observed for pre-dialysis diastolic BP (Fig. S2B).

After 12 months of follow-up, the prescription of antihypertensive drugs was similar in both groups: the mean number of tablets per day was 1.6 ± 1.3 vs 1.9 ± 1.3 (p = 0.13) in SDHD < 65 patients and 1.4 ± 1.3 vs 1.5 ± 0.8 (p = 0.98) in SDHD ≥ 65 patients. No difference in erythropoietin stimulating agent (ESA) dose was observed (“Supplementary files”).

At baseline, SDHD < 65 had higher mean albumin (3.7 ± 0.6 vs 3.4 ± 0.4 g/dL, p = 0.04) and lower mean pre-albumin levels (0.25 ± 0.1 vs 0.38 ± 0.1, *p* ≤ 0.001) compared to SDHD ≥ 65 (Fig S3A). Albumin levels in more than half of the SDHD ≥ 65 were below 3.5 g/dL. After 12 months of follow-up, albumin increased up to 3.9 ± 0.5 g/dL in SDHD < 65 but did not reach statistical significance. The mean pre-albumin level remained stable. No benefit of SDHD on nutritional parameters was observed in the SDHD ≥ 65 group, as albumin and pre-albumin levels remained stable during the first year, but at lower levels compared to SDHD < 65_._

Mean serum phosphate levels decreased in all patients during follow-up, but mainly in SDHD ≥ 65: 1.20 ± 0.3 mmol/L vs 1.48 ± 0.5 mmol/l in SDHD < 65, p = 0.09 (Fig. S3C).

The mean number of vascular events per patient per year (angiography-requiring procedure) was similar between the SDHD < 65 and SDHD ≥ 65 group (0.9 ± 1.8 vs 0.70 ± 1, *p* = 0.89).

The SDHD ≥ 65 patient group was matched to a control group, which included 49 prevalent HD patients ≥ 65 years old, with at least 24 months of dialysis. Demographic characteristics are shown in S2. Eight patients from the SDHD ≥ 65 group died, most of them of cardiovascular events (n = 6). The 24-month survival rate of SDHD ≥ 65 patients was not statistically different compared to the controls (67 vs 74%, p = 0.12) (Fig. 1).

**Fig. 1:**
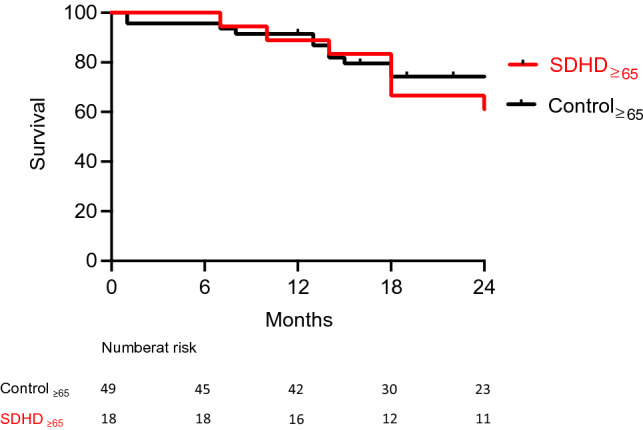
Two-year survival curves of elderly patients treated by in-center short daily hemodialysis. The graph depicts the survival curves up to 24 months after commencing short daily hemodialysis (SDHD) for patients older than 65 years (SDHD ≥ 65) in red, and the control group in black. The difference in survival between SDHD ≥ 65 and control > 65 did not reach statistical significance (p = 0.13, log-rank test) (colour figure online)

Two distinctive features of our elderly patients should be noted. Firstly, they had an altered baseline nutritional status including persistently low albumin and pre-albumin levels. Whilst SDHD controlled hyperphosphatemia in younger patients, in the elderly it appeared to promote hypophosphatemia, which has been shown to be an independent risk factor of mortality [2]. In our population, hypophosphatemia may be more indicative of the malnutrition status than of the efficacy of SDHD on phosphate removal. Secondly, more than half of our elderly group had a systolic BP below 120 mmHg which is also an independent risk factor of mortality [3]. This low BP could be due to diffuse arteriosclerosis and altered cardiac function. The comparable survival rate between the SDHD ≥ 65 and the control group should be interpreted cautiously. Indeed, we cannot exclude that the survival rate of SDHD ≥ 65 patients might have been worse if they had not been switched to SDHD.

Our study has several limitations. It is single-center, retrospective and involves a small number of patients. The comparison using a historical control group may have introduced bias, as both populations cannot be matched according to their dialysis vintage. Data regarding Quality of Life were not available. However, to our knowledge, this is the first study to focus on the outcome of elderly patients undergoing in-center SDHD.

Overall, our study demonstrated that SDHD for patients ≥ 65 years old is feasible, provides a clinical benefit regarding IDWG and should be considered an option for this fragile population.

## Supplementary Information

Below is the link to the electronic supplementary material.

Supplementary file1 (DOCX 136 KB)

Supplementary file2 (DOCX 16 KB)
