# Supplementary material for: Clinical outcome of short daily hemodialysis in the elderly
Source: J Nephrol. 2021 Aug 28;34(6):2171–2. doi: 10.1007/s40620-021-01148-1 (PMC8611047; doi:10.1007/s40620-021-01148-1)
Supplement: Supplementary file 1 — Supplementary file1 (DOCX 136 KB) [file 40620_2021_1148_MOESM1_ESM.docx]

**Supplementary Figure 1: Effect of short daily hemodialysis on fluid balance.**

The graph shows mean interdialytic weight gain (IDWG) before (M-3) and after short daily hemodialysis (SDHD) initiation, for SDHD≥65 (red) and SDHD<65 (blue). Data are expressed as mean ± SD. * *P*<0.05; ***P*=0.01; ns, non-significant (Mann Whitney or Wilcoxon test)

**Supplementary Figure 2. Control of predialysis systolic blood pressure after short daily hemodialysis (SDHD).**

The graph shows mean predialysis systolic blood pressure (sBP) at the start (M0) of short daily hemodialysis (SDHD) and at one year follow-up (M12), for SDHD≥65 (red), SDHD<65 (blue).

Data are expressed as mean ± SD. *p<0.05, ** p<0.01, ***p<0.001, ns: non-significant (Mann-Whitney test)

**Supplementary Figure 3**. **Evolution of biochemical parameters in short daily hemodialysis (SDHD) patients.**

The graph shows: (A) serum albumin, (B) pre-albumin, and (C) serum phosphate levels for SDHD≥65 (red), SDHD<65 (blue) at short daily hemodialysis start (M0), 6 (M6) and at 12 months (M12) follow-up. Data are expressed as mean ± SD. *p<0.05, ** p<0.01, ***p<0.001, ns: non-significant (Mann-Whitney test)

|  | **total (n=37)** | SDHD<65 **(n=19)** | SDHD≥65 **(n=18)** | **p-value** |
| --- | --- | --- | --- | --- |
| **Age (years, mean)** | 65±14 | 45±12 | 76±7 | <0.001 |
| **Female/Male** | 13/24 | 6/13 | 7/11 | 0.6 |
| **Ethnic group, n (%)** |  |  |  |  |
| Caucasian | 21 (57%) | 9 (47%) | 12 (67%) | 0.68 |
| Maghrebian | 10 (27%) | 6 (32%) | 4 (21%) |  |
| African | 5 (14%) | 4 (21%) | 1 (6%) |  |
| Other | 1 (3%) | 0 (3%) | 1 (6%) |  |
| **Nephropathy, n (%)** |  |  |  |  |
| Glomerulopathy | 23 (67%) | 14 (86%) | 9 (50%) | 0.30 |
| *Diabetes | 7 (43%) | 2 (11%) | 5 (28%) |  |
| TIN | 6 (16%) | 3 (16%) | 3 (17%) |  |
| Vascular | 5 (14%) | 1 (6%) | 4 (22%) |  |
| Other | 2 (6%) | 1 (6%) | 2 (11%) |  |
| Hypertension  Diabetes  Coronary Artery Disease  Atrial Fibrillation  PAD  History of stroke  History of cancer  BMI  Previous kidney transplant  Charlson Index | 24 (65%)  10 (27%)  13 (55%)  13 (35%)  12 (32%)  4 (11%)  14 (38%)  25+/5  17 (46%)  5.7+/2.9 | 12 (63%)  4 (21%)  4 (21%)  3 (16%)  2 (11%)  1 (6%)  9 (47%)  25+/6  13 (68%)  3.5+/1.4 | 12 (67%)  6 (33%)  9 (50%)  10 (56%)  10 (56%)  3 (17%)  5 (28%)  25+/-5  4 (22%)  8.1+/2.0 | 0.8  0.50  0.06  0.01  <0.01  0.3  0.2  1.00  0.005  <0.01 |
| **Dialysis parameters**  HDF/HD  Dialysis vintage before SDHD (months)  AVF, n (%)  **Indication of SDHD**  Patient request  Medical decision | 33/4 (89%)  26 (13-78)  32 (86%)  15 (41%)  22 (59%) | 19/0 (100%)  53 (10-158)  18 (95%)  11 (58%)  8 (42%) | 14/4(78%)  26 (18-47)  14 (78%)  4 (22%)  14 (78%) | 0.04  0.55  0.2  0.03 |
| **Clinical parameters**  sBP (mmHg)  dBP (mmHg)  IDWG (kg) mean,SD  IDWG (kg) median IQR | 134±22  71±16  1.93±0.9  1.9 (1.5-2.1) | 141±20  79±15  1.95±0.9  2 (1.3-2.8) | 127±23  64±14  1.92±1.1  1.9 (1.5-2) | 0.04  0.003  0.9  0 |
| **Biological parameters at M0**  Hemoglobin (g/dl)  Ferritin (ng/ml)  Calcium (mmol/l)  Phosphorus (mmol/l)  PTH (ng/ml)  Alkaline phosphatase (UI/L)  Albumin (g/dL)  Albumin <3.5 g/Dl (N)  Pre-albumin (g/dl)  25-OH vitamin D (ng/ml)  CRP (mg/l) | 11.5±1.5  388±283  2.20±0.20  1.49±0.50  347±218  103±62  3.6±0.6  15 (40%)  0.32±0.1  30±15  8±9 | 11.7±1.7  365±296  2.17±0.21  1.66±0.51  344±256  91±47  3.7±0.6  5 (16%)  0.38±0.1  26±11  7±9 | 11.3±1.3  415±272  2.24±0.20  1.29±0.42  350±172  108±53  3.4±0.4  10 (56%)  0.25±0.1  35±11  9.3±9 | 0.51  0.43  0.36  0.04  0.61  0.26  0.04  0.09  <0.001  0.33  0.10 |
| **Treatment**  RAAS Blockers  Betablockers  Calcium blockers  Diuretics  N (Mean)  Statin | 8 (22%)  20 (54%)  12 (32%)  20 (54%)  1.7±1.1  21 (57%) | 7 (37%)  9 (47%)  11 (58%)  7 (37%)  1.9±1.3  10 (53%) | 1 (6%)  11 (61%)  1 (6%)  13 (72%)  1.5±0.8  11 (61%) | 0.04  0.4  <0.001  0.03  0.27  0.6 |
| ESA (IU/kg/week)  vi.v. Iron (mg/month) | 90 (25-179)  100 (0-400) | 99 (60-176)  160 (50-400) | 66 (16-175)  150 (0-400) | 0.34  0.34 |

**Supplementary Table 1**. Patient characteristics and dialysis parameters.

Table represents data of the total population (N=37) and the sub-groups according to age < 65 years old (= SDHD<65), or ≥ 65 years old (SDHD≥65).

TIN: Tubulointerstitial nephropathy; PAD: peripheral arterial disease; BMI: body mass index; RAASi: Renin-angiotensin aldosterone system Inhibitors; HDF: Hemodiafiltration; HD vintage: hemodialysis Vintage; AVF: arteriovenous fistula; IDWG: intradialytic weight gain in kg; UFR: ultrafiltration rate in ml/h, sBP: systolic Blood Pressure; dBP: diastolic Blood Pressure. PTH: Parathyroid hormone; SDHD: Short Daily HemoDialysis

All continuous values are expressed as mean ± SD standard deviation; except for HD Vintage, IDWG, ESA and i.v. iron expressed as median and IQR (InterQuartile Range)

|  | **Controls (49)** | SDHD≥65 **(n=18)** | **p-value** |
| --- | --- | --- | --- |
| **Age (year, median)** | 74 (65-95) | 78 (66-85) | 0.8 |
| **Females/Males** | 26/23 | 7/11 | 0.3 |
| **Ethnic group, n (%)** |  |  |  |
| Caucasian | 31 (63%) | 12 (67%) | 0.7 |
| Maghrebian | 12 (24%) | 4 (22%) |  |
| African American | 5 (10%) | 1 (6%) |  |
| Other | 1 (2%) | 1 (6%) |  |
| **Nephropathy, n (%)** |  |  |  |
| Glomerulopathy | 19 (39%) | 9 (50%) | 0.7 |
| *Diabetes | 15 (31%) | 5 (28%) |  |
| TIN | 10 (20%) | 3 (17%) |  |
| Vascular | 14 (29%) | 4 (22%) |  |
| Other | 6 (12%) | 3 (9%) |  |
| Hypertension  Diabetes  Coronary Artery Disease  Atrial Fibrillation  PAD  History of stroke  History of cancer | 40 (82%)  25 (51%)  16 (33%)  12 (25%)  11 (22%)  6 (12%)  7 (14%) | 12 (67%)  6 (33%)  9 (50%)  10 (56%)  10 (56%)  3 (17%)  5 (28%) | 0.2  0.2  0.2  0.02  0.01  0.7  0.3 |
| **Type of** **epuration**  * HDF  *Hemodialysis  IDWG (Kg) mean, SD  IDWG (Kg) median, IQR | 42 (86%)  7 (14%)  1.3±0.9  1 (0.5-1.8) | 14 (68%)  4 (22%)  1.92±1.1  1.9 (1.5-2) | 0.2  0.69 |
| **Treatment**  Betablockers  RAAS blockers  Calcium blockers  Diuretics  N (mean) | 26 (53%)  21 (43%)  15 (31%)  33 (67%)  1.8±1.3 | 11 (61%)  1 (6%)  1 (6%)  12 (67%)  1.4±0.8 | 0.6  <0.001  0.05  1.00  0.43 |

**Supplementary Table 2**. Control and SDHD≥65 characteristics and dialysis parameters.

Table 2 represents data of the control (N=48) and SDHD ≥ 65-year-old patients.

TIN: Tubulointerstitial nephropathy; PAD: peripheral arterial disease; BMI: body Mass Index; RAASi: Renin-angiotensin aldosterone system Inhibitors; HDF: Hemodiafiltration; HD vintage: hemodialysis Vintage; IDWG: intradialytic weight gain in kg; UFR: Ultrafiltration rate in ml/h.

All continuous values are expressed as mean ± SD standard deviation; except for HD Vintage, IDWG expressed as median and IQR (InterQuartile Range)

SDHD: Short Daily HemoDialysis
