# Supplementary material for: Clinical outcome of short daily hemodialysis in the elderly
Source: J Nephrol. 2021 Aug 28;34(6):2171–2. doi: 10.1007/s40620-021-01148-1 (PMC8611047; doi:10.1007/s40620-021-01148-1)
Supplement: Supplementary file 2 — Supplementary file2 (DOCX 16 KB) [file 40620_2021_1148_MOESM2_ESM.docx]

**MATERIALS AND METHODS**

**Patient population:**

This multicenter, observational, and historical study, enrolled adult HD patients (≥ 18 years old) undergoing short daily hemodialysis (SDHD) in 2 dialysis centers (AURA Paris Plaisance, Paris and AURA Saint-Ouen) in France from January 2012 to December 2018.

Inclusion criteria were the following; HD patient > 6 months before the switch to SDHD either 5x2h30 or 6x2h sessions per week. SDHD was initiated at the patient’s request (to maintain a social life or job) or by medical decision (excessive IDWG, cardiac failure, frequent intradialytic hypotension).

Patient were divided into 2 groups according to the median age of 65-years: SDHD_<65_ and SDHD_≥65_.

Focusing on elderly SDHD outcome, we select a control group of HD patients, on thrice weekly hemodialysis, that was matched on age and dialysis vintage. The latter included 51 prevalent HD patients ≥ 65 years old, with at least 24 months of dialysis. We chose 24 months because it represents the median dialysis vintage of SDHD_≥65_ patients before the switch to SDHD. Due to the small population, adjustment for other comorbidities was not statistically feasible.

**Clinical and biological data:**

We collected patient epidemiological and laboratory data using our Medical informatics record system (Hemodial, PHP development, France and Medial, Nantes, France).

Demographic data collected were: age, sex, ethnicity, initial nephropathy, diabetes, coronary artery disease (CAD), atrial Fibrillation, Arterial hypertension, peripherical artery disease (PAD), history of stroke, history of cancer, Body Mass Index (BMI), HD vintage, HD technique (hemodiafiltration, or hemodialysis), dialysis time, dry weight and interdialytic weight gain (IDWG), ultrafiltration rate (UFR), systolic and diastolic blood pressure (BP) pre/post dialysis (measured by the nurse and recorded in our medical record), the use of anti-hypertensive drugs (beta-blocker, renin angiotensin aldosterone system [RAAS] blocker, calcium channel blocker), Intravenous (IV) Iron and Erythropoietin Stimulating Agent (ESA) doses.

IDWG and BP was defined as the mean of all IDWG and BP measurements for the week.

Biological parameters collected included: hemoglobin level, iron and ferritin levels, transferrin saturation (TSAT), C-Reactive Protein (CRP), phosphorus level, parathyroid hormone (PTH) concentration, 25-OH Vitamin D level, albumin and prealbumin level, and weekly weight-adjusted EPO doses (IU/kg/week).

**Statistical analysis of** **demographic or biological characteristic.**

Group statistics for continuous measures were reported as mean +/-SD for normally distributed measures or as median [IQR: Interquartile Range], otherwise; categorical measures were reported as count (percentage). Comparisons between patients were performed using a non-parametric test (Wilcoxon or Mann-Whitney test) or chi-square test, and Fisher’s exact test when appropriate. Graphic representation was performed with Prism Graphpad® software (version X.0; GraphPad, La Jolla, CA).

- All tests were two-sided and a p-value < 0.05 was considered significant. Statistical analyses were performed using R version 4.0.0 (R Foundation for Statistical Computing, Vienna, Austria). Survival analysis were performed with Prism Graphpad® software (version X.0; GraphPad, La Jolla, CA).
- **Ethical statement**

The study sponsor was AURA PARIS, and was approved by the institutional ethics committee (institutional review board number IRB00012157) and registered on the French health data hub. All patients were given information by their physician. The patients’ non-opposition to the use of their data for research was also collected in accordance with European regulations (General Data Protection Regulation).

**ESA and antihypertensive drug management**

Median weekly weight adjusted ESA (erythropoiesis stimulating agent) doses sightly decreased in the SDHD_<65_ group in a non-significant manner from 99 UI/kg/week (IQR: 60-176) to 71 UI/kg/week (IQR:18-143); p=0.06 but not in the SDHD_≥65_ group 56 UI/kg/week (IQR: 20-84) to 66 UI/kg/week (IQR: 16-175); p=0.44.

After one year of SDHD, the number of antihypertensive drugs was similar in both groups: the mean number of pills was 1.6±1.3 *vs* 1.9±1.3 (p=0.13) in SDHD_<65_ patients and 1.4±1.3 *vs* 1.5±0.8 (p=0.98), in SDHD_≥65_ patients.
